# Development of the AL-O-A Score for Delirium Screening in Acute Internal Medicine: a Monocentric Prospective Study

**DOI:** 10.1007/s11606-020-06502-w

**Published:** 2021-01-21

**Authors:** Gregor John, Vincent Bovet, Vincent Verdon, Hervé Zender, Jacques Donzé

**Affiliations:** 1 Department of Medicine, Neuchâtel Hospital Network, Neuchâtel, Switzerland; 2 Department of Internal Medicine, Geneva University Hospitals (HUG) and Geneva University, Geneva, Switzerland; 3 Department of Medicine, Neuchâtel Hospital Network, La Chaux-de-Fonds, Switzerland; 4 Department of Acute Medicine, Geneva University Hospitals (HUG), Geneva, Switzerland; 5 Department of Medicine, University Hospital of Lausanne, Lausanne, Switzerland; 6 Brigham and Women’s Hospital, Harvard Medical School, Boston, MA USA

**Keywords:** delirium, internal medicine, inpatients, adult, prospective study, score

## Abstract

**Background:**

Delirium occurs frequently in acute internal medicine wards and may worsen the patient’s prognosis; it deserves a fast, systematic screening tool.

**Objective:**

Develop a delirium screening score for inpatients admitted to acute internal medicine wards.

**Design:**

A monocentric prospective study between November 2019 and January 2020.

**Participants:**

Two hundred and seventeen adult inpatients.

**Main Measures:**

Within 48 h of hospital admission, physicians administered an index test to participants which explored potential predictors associated with the fluctuation of mental state, inattention, disorganised thinking and altered level of consciousness. On the same day, patients underwent a neuropsychological evaluation (reference standard) to assess for delirium. The score was constructed using a backward stepwise logistic regression strategy. Areas under the receiver operating curves (AUC) and calibration curves were drawn to calculate the score’s performance. The score was tested on subgroups determined by age, sex and cognitive status.

**Results:**

The AL-O-A score (“abnormal or fluctuating ALertness, temporospatial Orientation and off-target Answers”) showed excellent apparent (AUC 0.95 (95% CI 0.91–0.99)) and optimism-corrected discrimination (AUC 0.92 (95% CI 0.89–0.96)). It performed equally well in subgroups with and without cognitive impairment (AUC 0.93 (95% CI 0.88–0.99) vs 0.92 (95% CI 0.80–0.99)); in men and women (AUC 0.96 (95% CI 0.94–0.99) vs 0.95 (95% CI 0.89–0.99)); and in patients younger and older than 75 years old (AUC 0.98 (95% CI 0.95–0.99) vs 0.93 (95% CI 0.87–0.99)).

**Conclusions:**

A simple, 1-min screening test (AL-O-A score), even administered by an untrained professional, can identify delirium in internal medicine patients.

**Supplementary Information:**

The online version contains supplementary material available at 10.1007/s11606-020-06502-w.

## INTRODUCTION

Delirium (acute confusion) is frequent among patients admitted to acute care hospitals. In a meta-analysis, including patients mostly over 65 years old, its prevalence ranged from 19%–23%.^1^ However, very low and higher prevalence have been reported, depending on population age, the proportion of co-morbid diseases (mainly dementia, stroke, substance abuse) and clinical care settings.^2^

Delirium has a significant impact on health and prognosis, being associated with a prolonged hospital stay,^3^ faster cognitive decline, increased risk of fall,^3–5^ institutionalisation and death.^6^ Under-diagnosis is frequent and, in practice, only 30–60% of patients with delirium are appropriately diagnosed (outside studies or systematic screening).^2, 7, 8^ The estimated yearly costs attributable to delirium are USD 164 billion in the USA and USD 182 billion across 18 European countries.^9, 10^ Thus, delirium screening and prevention have major health and financial implications.

The Confusion Assessment Method (CAM)^11^ has long been the most-used bedside tool to diagnose delirium. Shorter versions or versions dedicated to specific clinical settings are also available, such as the CAM-ICU and the 3D-CAM.^12, 13^ Other brief screening tests (4 As Test, two-item bedside test, MOTYB-Spatial Span Forwards test) have been developed for specific populations (elderly, ICU, surgical/neurological patients).^12–16^ Nevertheless, because some need training, are time-consuming or restricted to specific settings, the distribution of many screening instruments is limited. Furthermore, most of these instruments perform poorly on patients with dementia. There remains a need to develop a brief, easy-to-use, reproducible screening tool, applicable to any admission to acute internal medicine wards. This study’s aim was, therefore, to develop a new screening score to identify inpatients with delirium at hospital admission.

## MATERIALS AND METHODS

To develop and internally validate a new delirium screening score, we performed a monocentric, prospective, observational study between 1 November 2019 and 6 January 2020. Physicians administered a questionnaire (index test) to all eligible patients within 48 h of their admission to the acute medical wards of a Department of Internal Medicine that included no neurology or psychiatric units. Patients underwent a neuropsychological examination to assess the presence or absence of delirium on the same day as they took the study questionnaire. Patients or their relatives gave written informed consent. These procedures followed the precepts of Good Clinical Practice and the Declaration of Helsinki. The Cantonal Ethics Committee, Vaud (CER-VD) approved the study. Reporting was performed according to the Transparent Reporting of a multivariable prediction model for Individual Prognosis Or Diagnosis (TRIPOD) statement.^17^

### Patient Inclusion and Predictors

All patients aged eighteen and over admitted during the period of interest were eligible. Patients whose planned length of stay was less than three days (mostly elective admissions), did not speak French or needing emergent care (e.g. oral intubation) were excluded.

A physician administered a 5-min index test to every patient within 48 h of admission, with a median delay of 21.8 h (IQR: 15.2–41.8). Potential predictors were selected based on clinical expertise and a literature review (GJ, VB and VV), with preference given to the simplest tests and questions.^8, 9, 11–16, 18–21^ The index test was composed of four subjective observations (abnormal alertness, fluctuation of mental state, illogical flow of ideas or unusually limited speech and off-target responses), two tasks (backwards digit and temporospatial orientation tests) and five questions (three on verbal logical reasoning, one to test associative visual agnosia and one to test for provoked confabulation) (Appendix methods). The study questionnaire also retrieved general information, comorbidities and prescribed medications.

All the physicians received a 45-min lesson on how to administer the test. The investigators visited every ward before and during the study to ensure that tests were performed correctly.

### Reference Standard Assessment

A clinical neuropsychologist (with more than 1 year’s experience) assessed the presence or absence of delirium in all included patients during a 15–30-min face-to-face interview based on the Diagnostic and Statistical Manual of Mental Disorders (DSM)-V criteria and the CAM method (Appendix methods).^22^ They also collected information from relatives and caregivers. In cases involving an undefined confusional state, doubts were resolved in consultation with two other neuropsychologists (more than 15 years of experience).

The study questionnaire and the neuropsychological evaluation were administered separately, with a median delay between the evaluations of 3.1 h (IQR: 1.9–5.8). The two paper-form documents were stored securely and were inaccessible through hospital medical records. Under specific recommendations not to interact, the neuropsychologist performed their evaluation at a time when the physician who had administered the study questionnaire was absent, thus leaving them blind to each other’s results.

### Statistical Analysis

We planned to include 200 patients in order to be comparable with a previous study in similar settings.^13^

We derived three scores. The first (main score) explored all potential predictors (subjective and objective), the second included objective predictors only (tasks and questions) and the third grouped predictors in one of the four CAM features. The CAM features were as follows: (1) fluctuation of mental state, (2) inattention, (3) disorganised thinking and (4) altered level of consciousness (Appendix methods).

Univariate analysis of every predictor variable collected in the study questionnaire was tested using logistic regression, with delirium being the dependent variable. Abnormal alertness (binary) and fluctuation of mental state (including alertness) were combined into a single binary predictor since they explored the same element and were collinear. Predictors associated with a *p* value < 0.2 were incorporated into a multivariable model only retaining informative variables (statistically significant) after using stepwise backwards selection methods. To ensure adequate predictor selection and estimate whether important predictors had been overlooked, the procedure was repeated 200 times in bootstrap samples of the same size as the original sample. Predictors retained in less than 50% of the bootstrapped models were discarded from the final model.^23^

The model’s accuracy was determined using discrimination and calibration. Calibration described how well the predicted probabilities fitted the observed probabilities, and discrimination tested whether the model correctly stratified patients at high and low risk of events.^24, 25^ To test the score’s discrimination, area under the receiver operating curves (AUC) were computed, and calibration was estimated using a visual inspection of the calibration curves. Brier scores, measuring model accuracy, were determined to assess overall model performance: lower scores reflecting greater accuracy. The three scores’ performances were compared using the full original dataset and subgroups of patients, dichotomised by cognitive impairment as documented on their medical charts (Appendix methods), sex or age (median), according to the nonparametric approach proposed by Delong et al.^26^

Since scores are over-fitted in the developed dataset, a bootstrap method was used to quantify the models’ optimism (internal validity) and correct their performance. This technique has been advocated as the best method for calculating internal validity.^25^ We fitted a logistic regression model to each bootstrapped sample, using the same backwards procedure used in the original dataset (to create the score). Optimism was then measured by subtracting the performance observed in the original sample from the apparent performance measured in the bootstrap sample. This procedure was averaged over 1000 repetitions. The bootstrap-corrected AUC was computed by subtracting the averaged optimism from the original AUC.

To create an easy-to-use score, we transformed each predictor’s beta coefficient into an integer (or half point). The score was then divided into categories. Category cut-offs were chosen in order to have a sensitive score and only a few patients in the intermediate category. We calculated sensitivity (Se), specificity (Sp), positive and negative predictive values (PPV and NPV) and positive and negative likelihood ratios (LR+ and LR−) for those cut-offs. Two independent raters administered the index test to 30 patients on the same day (test–retest). Inter-rater agreement was tested using Cohen’s kappa correlation for binary predictors and weighted kappa for ordinal predictors.

There were no missing values. All analyses were performed using Stata statistical software, version 12.0 (StataCorp LP, College Station, TX, USA).

## RESULTS

**Fig. 1:**
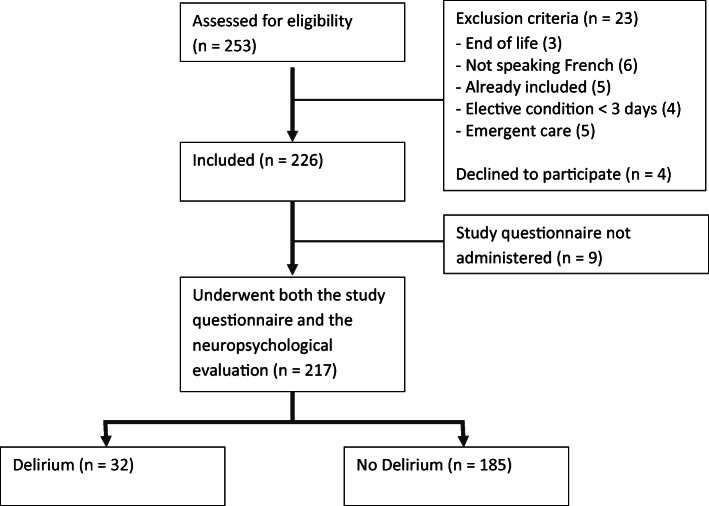
Study flowchart.

During the study period, 217 participants admitted to the acute medical ward underwent both the study questionnaire and a dedicated neuropsychological evaluation (Fig. 1). Ages ranged from 19–104 years old, with a median age of 76 (IQR: 66–85). Men (*n* = 108) and women (*n* = 109) were equally represented.

The neuropsychologist determined that 32 admitted patients (16%) had delirium. Six patients needed reassessment by two other neuropsychologists for a definitive diagnosis (3 with and 3 without delirium). The univariate analyses associated all of the study questionnaire’s predictors with delirium (Table 1). The subjective predictors and the five questions were specific but had poor sensitivity. Tasks were sensitive, and their specificity increased with the number of mistakes but with a decreasing sensitivity. The backwards digit and temporospatial orientation tests were the two most discriminative single items, with AUC of 0.82 (0.73–0.90) and 0.87 (0.79–0.94), respectively (Table 1 in the Appendix section).

**Table 1:** Population Characteristics and Study Questionnaire Predictors Distribution Among Patients With and Without Delirium

Variables	Complete sample (217)	Delirium (*n* = 32)	No delirium (*n* = 185)	*p* value
**Primary diagnosis:**				
- Infection	57 (26%)	15 (47%)	42 (23%)	0.16
- Heart failure	41 (19%)	4 (12%)	37 (20%)	
- Cancer	24 (11%)	1 (3%)	23 (12%)	
- Fall/syncope	17 (8%)	2 (6%)	15 (8%)	
- Toxic	13 (6%)	2 (6%)	11 (6%)	
- Rheumatology	12 (5%)	2 (6%)	10 (5%)	
- Anaemia	11 (5%)	1 (3%)	10 (5%)	
- Pulmonary disease	10 (5%)	0	10 (5%)	
- Metabolic disease	10 (5%)	1 (3%)	9 (5%)	
- Thrombosis	7 (3%)	0	7 (4%)	
- Others	15 (7%)	4 (12%)	11 (6%)	
**Co-morbid conditions/secondary diagnosis**				
Ischemic heart disease	53 (24%)	12 (37%)	41 (22%)	0.07
Heart failure	90 (41%)	17 (53%)	73 (39%)	0.15
Peripheral arterial diseases	19 (9%)	2 (6%)	17 (9%)	0.74
Abdominal aneurism	8 (4%)	2 (6%)	6 (3%)	0.35
Atrial fibrillation	63 (29%)	12 (37%)	51 (28%)	0.29
Pulmonary embolism	33 (15%)	6 (19%)	27 (15%)	0.55
High blood pressure	140 (65%)	24 (75%)	116 (63%)	0.18
Cerebrovascular disease	43 (20%)	12 (38%)	31 (17%)	0.07
Parkinson disease	6 (3%)	1 (3%)	5 (3%)	0.89
Mild cognitive impairment	39 (18%)	8 (25%)	31 (17%)	< 0.001
Moderate-to-severe cognitive impairment*	45 (21%)	16 (50%)	29 (16%)	
Chronic obstructive pulmonary disease	40 (18%)	4 (12%)	36 (19%)	0.46
Asthma	8 (4%)	2 (6%)	6 (3%)	0.35
Cirrhosis	24 (11%)	2 (6%)	22 (12%)	0.52
Gastro-oesophageal reflux disease	41 (19%)	4 (12%)	37 (20%)	0.46
Chronic renal disease				0.03
KDIGO3	34 (16%)	10 (31%)	24 (13%)	
KDIGO4–5	15 (7%)	3 (9%)	12 (6%)	
Acute renal disease				0.01
KDIGO1	53 (24%)	11 (34%)	42 (23%)	
KDIGO2	10 (5%)	2 (6%)	8 (4%)	
KDIGO3	5 (2%)	3 (9%)	2 (1%)	
Diabetes	72 (33%)	7 (22%)	42 (23%)	0.92
Rheumatologic/orthopaedic	78 (36%)	18 (56%)	60 (32%)	0.01
Oncologic disease	66 (30%)	7 (22%)	59 (32%)	0.30
Denutrition	49 (23%)	18 (56%)	54 (29%)	0.01
Alcohol at risk	37 (17%)	4 (12%)	33 (18%)	0.55
Toxic abuse	9 (4%)	1 (3%)	8 (4%)	0.99
Tobacco				0.64
Former	22 (10%)	2 (6%)	20 (11%)	
Active	43 (20%)	5 (16%)	38 (21%)	
Psychiatric disease				
Mood disorder	63 (29%)	12 (37%)	51 (28%)	0.26
Psychotic disease	8 (4%)	3 (9%)	5 (3%)	0.10
**Subjective observation**				
Normal alertness vs				< 0.001
Drowsy	11 (5%)	8 (25%)	3 (2%)	
Agitated	9 (4%)	7 (21%)	2 (1%)	
Fluctuation of mental status	27 (12%)	18 (56%)	9 (5%)	< 0.001
Illogical flow of ideas	30 (14%)	18 (56%)	12 (6%)	< 0.001
Off-target responses	24 (11%)	18 (56%)	6 (3%)	< 0.001
**Tasks**				
Backwards digit test				
1 mistake	73 (34%)	3 (9%)	70 (38%)	0.93
2 mistakes	31 (14%)	3 (9%)	28 (15%)	0.33
3 or more mistakes	46 (21%)	23 (72%)	23 (12%)	< 0.001
Temporospatial orientation				
1 mistake	29 (13%)	5 (16%)	24 (13%)	0.005
2 mistakes	18 (8%)	6 (19%)	12 (6%)	< 0.001
3 or more mistakes	26 (12%)	17 (53%)	9 (5%)	< 0.001
**Questions**				
Q1	37 (17%)	13 (41%)	24 (13%)	< 0.001
Q2	51 (23%)	15 (47%)	36 (20%)	0.001
Q3	21 (10%)	13 (40%)	8 (4%)	< 0.001
Q4	10 (5%)	9 (28%)	1 (0.5%)	< 0.001
Q5	34 (16%)	11 (34%)	23 (12%)	0.003
Q1–5				
1 mistake	53 (24%)	5 (16%)	48 (26%)	0.84
2 mistakes	20 (9%)	5 (16%)	15 (8%)	0.03
3 mistakes	7 (3%)	4 (12%)	3 (2%)	0.001
4 or more mistakes	8 (4%)	7 (22%)	1 (0.5%)	< 0.001

*According to neuropsychological examinations, Mini-Mental State Examination (MMSE), Montreal Cognitive Assessments (MoCa) ≤ 20 or Clinical Dementia Rating (CDR) > 1 performed before or at least three months after an episode of delirium

*KDIGO*, Kidney Disease: Improving Global Outcomes

Q1: “Is it possible to walk from here (Switzerland) to New York?”

Q2: “Is one kilogram of lead heavier than one kilogram of feathers?”

Q3: “How many legs does a sheep that has lost one leg have?”

Q4: “What is this for?” (Showing the patient a watch, then a pen)

Q5: “What was Marilyn Monroe’s father’s shoe size?” (Unanswerable)

### Main Score

**Fig. 2:**
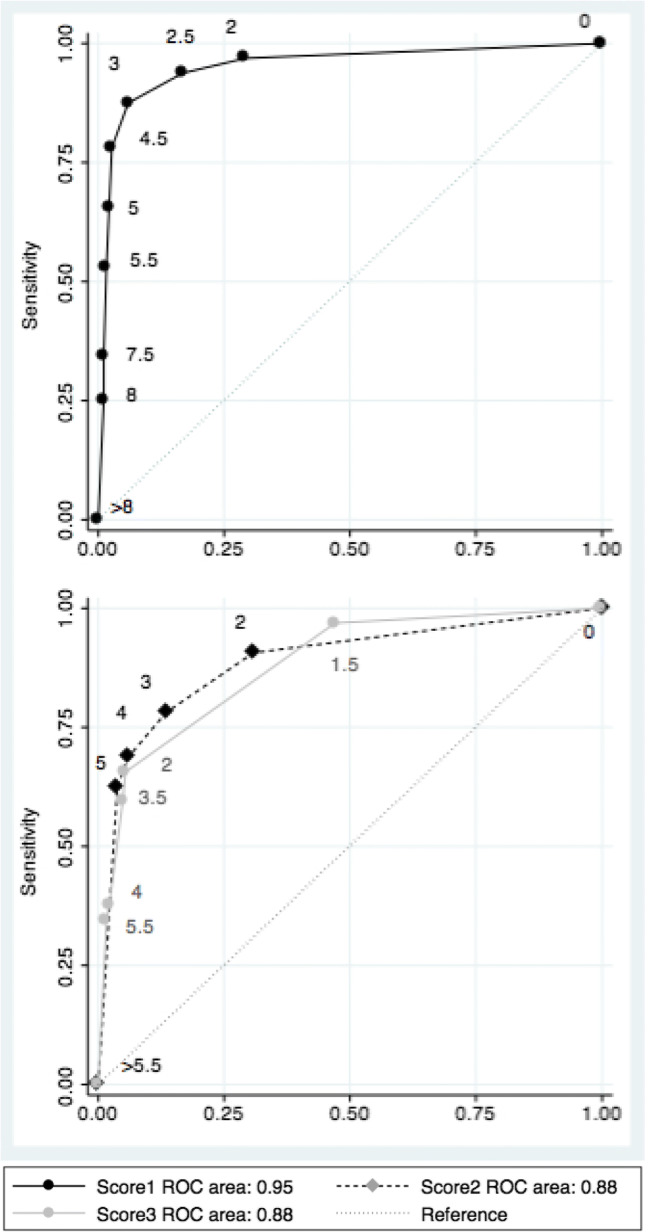
Receiver operating characteristic (ROC) curve for the three scores. Score 1: derived using all predictors; Score 2: derived using objective predictors only; Score 3: derived using the predictors grouped by features of the Confusion Assessment Method.

In the multivariable logistic regression, temporospatial orientation, non-normal/fluctuating alertness (binary), and off-target answers were incorporated into the final score (Table 2, Fig. 2). The model displayed excellent apparent and optimism-corrected performance (Table 3). The score performed equally well in subgroups of patients categorised by age, sex or cognitive status (Table 3, Table 2 in the Appendix section). Calibration on the calibration plots and according to the Brier score was excellent (Table 2, Fig. 1 in the Appendix section).

**Table 2:** Multivariable Models with Beta Coefficient and Simplified Scoring System

Variables	Coef. B (95%CI)	Simplified score
**Score 1—all predictors**		
Temporospatial orientation		
1 mistake	2.1 (0.5–3.8)	2
2 mistakes	2.4 (0.5–4.2)	2.5
3 or more mistakes	3.2 (1.5–4.8)	3
Abnormal alertness (or fluctuating mental status)	2.6 (1.3–3.9)	2.5
Off-target responses	2.4 (0.7–4.0)	2.5
**Score 2—objective predictors only**		
Temporospatial orientation		
1 mistake	1.8 (0.3–3.2)	2
2 mistakes	2.7 (1.3–4.2)	3
3 or more mistakes	2.9 (1.5–4.4)	3
3 or more mistakes in backwards digit test	1.9 (0.7–3.0)	2
**Score 3—CAM-based grouped predictors**		
Feature 1	1.9 (0.7–3.2)	2
Feature 2	-	-
Feature 3	1.6 (0.4–2.9)	1.5
Feature 4	2.0 (0.6–3.4)	2

**Table 3:** Apparent and Optimism-Corrected Performance of the Scores in the Original Dataset and in Subgroups

	Score 1—All predictors	Score 2—Objective predictors only	*p* value*	Score 3—CAM-based grouped predictors	*p* value†
Overall AUC	0.95 (0.91–0.99)	0.88 (0.82–0.95)	0.02	0.88 (0.82–0.94)	< 0.001
AUC in patients					
With cognitive impairment (*n* = 83)	0.93 (0.88–0.99)	0.86 (0.77–0.94)	0.04	0.83 (0.75–0.92)	0.01
Without cognitive impairment (*n* = 134)	0.92 (0.80–0.99)	0.83 (0.67–0.99)	0.19	0.89 (0.74–0.99)	0.06
AUC in patients					
Younger than 75 years (*n* = 103)	0.98 (0.95–0.99)	0.95 (0.91–0.99)^‡^	0.29	0.90 (0.82–0.98)	0.03
Over 75 years (*n* = 114)	0.93 (0.87–0.99)	0.84 (0.74–0.95)^‡^	0.04	0.87 (0.78–0.95)	0.01
AUC in					
Women (*n* = 108)	0.95 (0.89–0.99)	0.90 (0.82–0.98)	0.09	0.85 (0.77–0.94)	< 0.001
Men (*n* = 109)	0.96 (0.93–0.99)	0.88 (0.76–0.99)	0.15	0.93 (0.87–0.99)	0.14
Brier score	0.050	0.0724	-	0.0798	-
Optimism	0.03	0.01	-	0.01	-
Optimism-corrected overall AUC	0.92 (0.89–0.96)	0.87 (0.81–0.94)	-	0.87 (0.81–0.93)	-

^*^p value for the difference in AUC between score 1 and score 2

^†^*p* value for the difference in AUC between score 1 and score 3

^‡^*p* = 0.06 for subgroup difference (younger vs older patients)

A simplified score (Table 4) of 0 showed excellent negative predictive value (99%), with 132 patients (61%) scoring 0. A score of 4.5 or higher showed a positive predictive value of a definite diagnosis of delirium of 83%, with 30 patients (14%) scoring 4.5 or higher. Fifty-five patients (25%) fell into the intermediate category, with a delirium prevalence of 11% (Table 4).

**Table 4:** Categorisation of the Three Scores into Low, Intermediate and High Probability. Sensitivity (Se), Specificity (Sp), Negative and Positive Predictive Values (NPV, PPV) and positive and negative Likelihood Ratios (LR+, LR−) Are Given for Different Cut-Off Points Considering a Positive Score (This and Higher Values Being Positive). The First and Second Cut-Off Points Separate Low and Intermediate Categories, and Intermediate and High Categories, Respectively. All Possible Cut-Off Points, Their Corresponding LR and Post-Test Probabilities Can Be Found in Table 1 in the Appendix Section

Probability	Delirium, *n* (%)	Cut-off points	Se	Sp	NPV	PPV	LR+	LR−
**Score 1 (AL-O-A)**								
Low	1/132 (1%)							
Intermediate	6/55 (11%)	2	97% (4–100)	71% (64–77)	99% (96–100)	36% (26–48)	3.3 (2.6–4.2)	0.04 (0.01–0.30)
High	25/30 (83%)	4.5	78% (60–91)	97% (94–99)	96% (92–98)	83% (65–94)	28.9 (11.9–70)	0.22 (0.12–0.43)
**Score 2**								
Low	3/131 (2%)							
Intermediate	7/53 (13%)	2	91% (75–98)	69% (62–76)	98% (93–99)	34% (24–45)	2.9 (2.3–3.8)	0.13 (0.05–0.40)
High	22/33 (67%)	4	69% (50–84)	94% (90–97)	95% (90–97)	66% (48–82)	11.6 (6.2–21.5)	0.33 (0.20–0.56)
**Score 3**								
Low	1/99 (1%)							
Inter- mediate	9/87 (10%)	1.5	97% (84–99)	53% (45–60)	99% (94–100)	26% (19–35)	2.1 (1.8–2.4)	0.06 (0.01–0.41)
High	21/31 (68%)	2	66% (47–81)	95% (90–97)	94% (90–97)	68% (49–83)	12.1 (6.3–23.3)	0.36 (0.22–0.59)

*LR*, likelihood ratio; *NPV*, negative predictive value; *PPV*, positive predictive value; *Se*, sensitivity; *Sp*, specificity

The results of the two secondary scores are shown in Fig. 2, Tables 1–4 and the Appendix section.

### Inter-Rater Reliability

Inter-rater agreement was moderate or good for most predictors (73–93%; kappa 0.45–0.81) (Table 4 in the Appendix section). Of note, inter-rater agreement was only moderate for the backwards digit test (74% agreement; weighted kappa 0.45). Subjective predictors showed good to very good inter-rater performance.

Agreement for the low versus other categories of the main score was good (87%; kappa 0.72) but poor for the score based on objective predictors (63%; kappa 0.32). Inter-rater reliability was good for the main score’s overall classification (88%; kappa 0.70) and moderate for the score based on objective predictors (75%; kappa 0.46) (Table 4 in the Appendix section).

## DISCUSSION AND CONCLUSION

The AL-O-A score (ALertness, Orientation, off-target Answers) for delirium screening in internal medicine wards is short (three items) and easy to administer. It displays excellent discriminative performance, even in subgroups categorised by age, sex and cognitive status.

Patients with delirium have a twofold increased risk of death and a two-to-three-fold increased risk of institutionalisation.^6^ Undiagnosed delirium has been associated with longer hospital length of stay and worse cognitive performance.^3^ Furthermore, delirium persists in up to one quarter of patients at 6 months after discharge.^27, 28^ Diagnosis of delirium thus has a direct impact on hospitalisation and potentially influences care at discharge. Since clinical judgement is insufficient, delirium diagnosis requires a high index of suspicion.^2, 7^ To that end, the AL-O-A score was developed as a screening tool that could be administered to any admission, favouring sensitivity over specificity. The resulting score displays excellent negative predictive value, easily excluding two thirds of patients. Administered by untrained, junior physicians in the present study, the AL-O-A score could be quickly incorporated into the daily practice of many hospitals by physicians with very different experience levels. Since most of the items are part of standard admissions procedures in internal medicine settings, the systematic application of the score would not extend the time dedicated to a medical workup. Nevertheless, further studies should explore this strategy’s clinical and economic relevance.

The AL-O-A score lacks several essential criteria for a definite diagnosis of delirium as listed in the DSM-V.^22^ Notably, the score does not capture whether a disturbance develops over a short period (usually hours/days) or fluctuates over time, and those features are essential to distinguishing delirium from dementia.^9^ Furthermore, to diagnose delirium, the physician must acknowledge that disturbances are not better explained by another neurocognitive disorder and are not occurring in the context of a severely reduced arousal level, such as coma. The score assumes that disturbances are the direct physiological consequence of another medical condition, including substance or toxin intoxication or withdrawal.^22^ Although the AL-O-A score was designed as a screening strategy and lacks several DSM-V criteria, its positive predictive value (83%) for high-level probabilities (score 4.5 or higher) is good enough to alter immediate medical management. The probability is sufficient to initiate a thorough workup looking for aetiology, correct associated factors and include these patients in preventive programmes (e.g. against in-hospital falls, the Hospital Elder Life Program (HELP) and others).^29^

The best single-item tests for delirium screening were orientation and attention tests. The backwards digit (AUC 0.82) and temporospatial orientation (AUC 0.87) tests were the most discriminative. Making one mistake (in either test) had a sensitivity of around 90% and a negative predictive value of > 90%. Making three or more mistakes had a specificity around 90% and a positive predictive value from 50–65%. This observation was in line with the study by Fick et al.^30^ However, the score constructed with objective predictors only, including both the orientation and attention tests, had no advantages over the main score and performed slightly less well, with moderate inter-rater reliability.

The score based on CAM features was the least discriminating and far more complex. It was therefore worthless in a rapid screening strategy.

Several tools exist for inpatient delirium diagnosis. The closest to the AL-O-A score—when considering the reference standard used and the items that compose the final score—is the 4 As Test (4AT).^14, 15^ This shares *alertness* and some items concerning *orientation* present in the AL-O-A score. The 4AT also tests attention through its *months of the year backwards* (MOTYB) test and acknowledges delirium’s acute and fluctuating courses. However, the 4AT was developed in a geriatric population aged 70 and over. It performs as well as the AL-O-A score at the 4.5 points cut-off (sensitivity of 76% vs 78%, and specificity of 94% vs 97%, respectively).^15^ However, the AL-O-A score displays better sensitivity at the 2 points cut-off (97% sensitivity and 71% specificity). Thus, a patient with normal alertness, giving no off-target answers and able to identify the date and their location is unlikely to be confused. The 3D-CAM is a structured assessment tool displaying 95% sensitivity and 94% specificity.^13^ It was derived among patients aged 75 years old or more. Although this widespread test performs well and takes far less time to administer than the complete CAM, it remains a complex tool that would be difficult to generalise as an initial screening test. The same research group developed an ultra-brief, two-item, bedside test for delirium composed of the MOTYB test and the “What day of the week is it?” test.^30^ This test has 93% sensitivity and 64% specificity among geriatric populations (one quarter suffering from dementia). O’Regan and colleagues administered attention tests (MOTYB and the Spatial Span Forwards test) to 265 adult inpatients (median age 69 years old) and found them to be sensitive.^16^ However, they did not formally assess delirium in all participants (excluding patients who passed both attention tests), which may have influenced the attention test’s sensitivity. Besides, they administered this as a 1-day study, including patients at very different stages of their diseases (new admissions and patients about to be discharged). Finally, the population was also composed of surgical and neurological patients. Thus, to the best of our knowledge, AL-O-A is the first score developed for any admission to an internal medicine ward, unrestricted by age or dedicated to at-risk patients.

The present study has a few limitations. First, not having restricted the study by age or to a priori at-risk patients resulted in low delirium prevalence, which affected the study’s power (few events per predictor) and could have inflated the score’s performance. However, its performance was similar in both the lowest (low prevalence) and highest age quartiles (high prevalence). Secondly, two of the score items are subjective. Nevertheless, many delirium scores include these items, with good inter-rater agreement.^12, 13, 15^ The two subjective predictors were discriminative even though untrained physicians administered the test. Furthermore, the test’s performance was constant over a variety of subgroups, indicating that the presence or absence of items was easy to assess, even in older patients or patients with cognitive impairment. In an effort to develop a more reproducible measure, we tested an alternative score, including objective predictors only, but it performed worse. Thirdly, because some of the score’s questions are close to those asked during the neuropsychological examination, there could be incorporation bias. However, the AL-O-A score is far simpler and less time-consuming; therefore, we believe that its usefulness has been demonstrated. Fourthly, although external validation is the preferred validation method whenever possible, no independent sample was available for study. We instead performed an internal validation.^17^ Among internal validation methods, bootstrapping is the recommended technique for correcting a model’s performance for optimism.^17^ Fifthly, the reference standard was based on classic, validated scales administered by an experienced neuropsychologist. However, neuropsychological evaluation for the diagnosis of delirium could have differed between raters. Finally, the test was developed and evaluated in a French-speaking Swiss population sample (the English version is provided in the Appendix section), and its performance might vary in other languages or cultures. Nevertheless, AL-O-A’s individual predictors can be found in several instruments validated in different languages with equivalent performance.^12, 13, 15^ Besides, the score was administered during the 48 h after admission, and patients with strokes, acute psychiatric diseases or post-surgery were not included. Thus, the score’s performance remains unknown in these patient groups or later after admission.

In conclusion, the AL-O-A score is fast, easy and performs well, even among older adults or patients with a cognitive impairment admitted to an internal medicine ward. Further studies are now needed to assess its clinical relevance as a systematic screening tool for any admission and its performance in other settings (external validation).

## Supplementary Information

ESM 1 (DOCX 324 kb)
