# Supplementary material for: Development of the AL-O-A Score for Delirium Screening in Acute Internal Medicine: a Monocentric Prospective Study
Source: J Gen Intern Med. 2021 Jan 21;36(7):1980–8. doi: 10.1007/s11606-020-06502-w (PMC8298741; doi:10.1007/s11606-020-06502-w)
Supplement: Supplementary file 1 — (DOCX 324 kb) [file 11606_2020_6502_MOESM1_ESM.docx]

Appendix

| **Appendix Methods**   - Predictors included in the study questionnaire. - How predictors were grouped into one of the four CAM features. - Neuropsychological examination - Cognitive impairment | Page 30-31 |
| --- | --- |
| **Results of the two secondary scores** | Page 32 |
| **Appendix Table 1**: Study questionnaire predictors and their association with delirium. | Page 33 |
| **Appendix Table 2**: Performance of the AL-O-A score stratified by age quartile and cognitive impairment severity. | Page 34 |
| **Appendix Table 3**: Likelihood ratios and post-test probabilities for the three scores at all cut-off points. | Page 35 |
| **Appendix Table 4**: Inter-ratter agreement for the study questionnaire predictors. | Page 36 |
| **Appendix Table 5**: Precipitating factors in patients with delirium. | Page 37 |
| **Appendix Figure 1**: Calibration curves for the three scores with LOWESS smoother. | Page 38 |
| **AL-O-A score** | Page 39-40 |

**Appendix Methods:**

Predictors included in the study questionnaire.

The subjective items included: 1) abnormal alertness (patient drowsy or agitated; yes or no); 2) fluctuation of mental state (alertness or level of attention; yes or no); 3) illogical flow of ideas or unusually limited speech (yes or no); 4) off-target responses (yes or no). The tasks included: 1) repeating three and four digits backwards (backwards digit test) (0, 1, 2, and 3 or more mistakes); 2) spatial and temporal orientation (“*What is the date today?*” (day, month and year), and “*Where are we?*” (state/city and type of building (hospital)), (0, 1, 2, or 3 or more mistakes). The backwards digit test starts with the repetition of a string of three-digits (e.g. 8, 5, 3) presented orally. Then the patient’s ability to recall of the digits backwards is tested (e.g. 3, 5, 8). The test is then repeated with a four-digit sequence (e.g. 1, 6, 9, 2). Any mistake in the digits given backwards (e.g. 7 instead of 5) or digit sequence (e.g. 3, 8, 5 instead of 3, 5, 8) for the 3-digit and 4-digit tests is added. The five questions were: a) *“Is it possible to walk from here (Switzerland) to New York?”* b) *“Is one kilogram of lead heavier than one kilogram of feathers?”* c) *“How many legs does a sheep that has lost one leg have?”* d) “*What is this for?*” (Showing the patient a watch, then a pen) e) “What was Marilyn Monroe’s father’s shoe size?” (Correct answer “I don’t know“).

How predictors were grouped into one of the four CAM features.

The CAM features were: 1) fluctuation of mental state (second observation on the study questionnaire), 2) inattention (first task and fourth observation on the study questionnaire), 3) disorganised thinking (second task, third observation and the logical question on the study questionnaire), and 4) altered level of consciousness (first observation on study questionnaire).

Neuropsychological examination

The neuropsychological evaluation was based on clinical observations and objective measures such as the Digit Span subtest from the *Wechsler Adult Intellectual Scale - 4th Edition* (2008) and items from a visual recognition cognitive assessment tool for French-speaking older adults (*Protocole d’Examen Cognitif de la Personne Agée-Lausanne*, 2006). Validated scales were also used to assess the level of consciousness (*Richmond Agitation Sedation Scale*, 2003) and the fluctuating course of mental state (*The Clinician Assessment of Fluctuation*, 2000).

Cognitive impairment

We explored the entire hospital database for the results of neuropsychological examinations, Mini-Mental State Examination (MMSE), Montreal Cognitive Assessments (MoCa) and Clinical Dementia Rating (CDR) performed before or at least three months after an episode of delirium. Six patients with delirium underwent their evaluation within one month of their acute confusion. However, five had an impaired cognitive status known before admission, confirmed by a previous evaluation and/or heteroanamnesis of their family members (found in the neuropsychological examination). We found no relevant cognitive impairment in the sixth patient, since no mention had been made in the prior discharge letter and no previous MMSE/MoCa were available (at a date sufficiently distant from the episode of delirium).

We further stratified cognitive state into: no cognitive impairment (MMSE/MoCa > 26, and/or no information in the medical database), mild cognitive impairment (MMSE/MoCa > 20 and ≤ 26, and/or CDR = 1, and/or noted “mild” in neuropsychological reports), and moderate-to-severe cognitive impairment (MMSE/MoCa ≤ 20, and/or CDR > 1, and/or noted “moderate” or “severe” in neuropsychological reports).

**Results for the score restricted to objective predictors**

When score construction was restricted to objective predictors, the temporospatial orientation and backwards digit tests were incorporated in the multivariate model (**Table 2**). Discriminative performance was good even after correction for optimism (**Table 3, Figure 2**). The model performed better for patients younger than 75 years old than for older patients, but the difference was not statistically significant (*p* = 0.06). Its performance was not as good as the main score’s, especially for older patients (**Table 3**). Calibration on the calibration plots and according to the Brier score was excellent (**Appendix Figure 1, Table 2**).

One hundred thirty-one patients (60%) had a simplified score of 0, with a negative predictive value of 98%. A score higher than 2 had a positive predictive value of 66% and was encountered in 33 patients (15%). Fifty-three patients (24%) were in the intermediate category (13% of delirium) (**Table 4**).

**Results for the score constructed from study questionnaire-based CAM predictors**

The last model using predictors grouped by CAM domain included three of the four domains (**Table 2**). It performed worse than the two other scores, except in men, but the difference was not statistically significant (**Table 2**). The score was adequately calibrated on the calibration plots and displayed good overall performance (low Brier score) (**Appendix Figure 1, Table 2**).

| **Appendix Table 1**: Study questionnaire predictors and their association with delirium. The table shows each predictor’s distribution among patients with and without delirium, the univariate logistic regression beta coefficient (Coef. B), sensitivity (Se), specificity (Sp) and the area under the receiver operating characteristic curve (AUC) for delirium diagnosis. | | | | | | | |
| --- | --- | --- | --- | --- | --- | --- | --- |
| Variables | No delirium (n = 185) | Delirium (n = 32) | Coef. B (95%CI) | *P*-value | Se | Sp | AUC |
| Subjective observation | | | | | | | |
| Normal alertness vs  Drowsy  Agitated | 3 (2%)  2 (1%) | 8 (25%)  7 (21%) | 3.5 (2.3–4.6) | < 0.001 | 47 | 97 | 0.72 (0.63–0.81) |
| Fluctuation of mental status | 9 (5%) | 18 (56%) | 3.2 (2.3–4.2) | < 0.001 | 56 | 95 | 0.76 (0.67–0.85) |
| Illogical flow of ideas | 12 (6%) | 18 (56%) | 2.9 (2.0–3.8) | < 0.001 | 56 | 94 | 0.75 (0.66–0.84) |
| Off-target responses | 6 (3%) | 18 (56%) | 3.6 (.6–4.7) | < 0.001 | 56 | 97 | 0.76 (0.68–0.85) |
| Tasks | | | | | | | |
| Backwards digit test  1 mistake  2 mistakes  3 or more mistakes | 70 (38%)  28 (15%)  23 (12%) | 3 (9%)  3 (9%)  23 (72%) | -0.1 (-1.7–1.5)  0.8 (-0.8–2.5)  3.1 (1.8–4.3) | 0.93  0.33  < 0.001 | 91  81  72 | 35  72  88 | 0.82 (0.73–0.90) |
| Temporospatial orientation  1 mistake  2 mistakes  3 or more mistakes | 24 (13%)  12 (6%)  9 (5%) | 5 (16%)  6 (19%)  17 (53%) | 1.9 (0.6–3.4)  2.9 (1.5–4.3)  4.2 (2.9–5.5) | 0.005  < 0.001  < 0.001 | 88  72  53 | 76  89  95 | 0.87 (0.79–0.94) |
| Questions | | | | | | | |
| Q1 | 24 (13%) | 13 (41%) | 1.5 (0.7–2.3) | < 0.001 | 41 | 87 | 0.64 (0.55–0.73) |
| Q2 | 36 (20%) | 15 (47%) | 1.3 (0.5–2.1) | 0.001 | 47 | 80 | 0.64 (0.54–0.73) |
| Q3 | 8 (4%) | 13 (40%) | 2.7 (1.7–3.7) | < 0.001 | 41 | 96 | 0.68 (0.59–0.77) |
| Q4 | 1 (0.5%) | 9 (28%) | 4.3 (2.2–6.4) | < 0.001 | 28 | 99 | 0.64 (0.56–0.72) |
| Q5 | 23 (12%) | 11 (34%) | 1.3 (0.5–2.2) | 0.003 | 34 | 88 | 0.61 (0.52–0.70) |
| Q1–5  1 mistake  2 mistakes  3 mistakes  4 or more mistakes | 48 (26%)  15 (8%)  3 (2%)  1 (0.5%) | 5 (16%)  5 (16%)  4 (12%)  7 (22%) | 0.1 (0.0–1.2)  1.3 (0.1–2.5)  2.7 (1.1–4.3)  4.3 (2.1–6.5) | 0.84  0.03  0.001  < 0.001 | 66  50  34  22 | 64  84  88  85 | 0.72 (0.61–0.82) |
| Q1: “Is it possible to walk from here (Switzerland) to New York?”  Q2: “Is one kilogram of lead heavier than one kilogram of feathers?”  Q3: “How many legs does a sheep that has lost one leg have?”  Q4: “What is this for?” (Showing the patient a watch, then a pen)  Q5: “What was Marilyn Monroe’s father’s shoe size?” (Unanswerable) | | | | | | | |

| **Appendix Table 2** Performance of the AL-O-A score stratified by age quartile and cognitive impairment severity. | | | | |
| --- | --- | --- | --- | --- |
|  | N | Delirium (%) | AUC | *P*-value |
| Age quartiles | | | | |
| <65 years | 52 | 3 (6%) | 0.98 (0.95-0.99) | 0.53 |
| 65-74 years | 51 | 7 (14%) | 0.97 (0.93-0.99) |  |
| 75-84 years | 57 | 7 (12%) | 0.94 (0.87-0.99) |  |
| >85 years | 57 | 15 (26%) | 0.93 (0.83-0.99) |  |
| Cognitive status | | | | |
| No cognitive impairment | 134 | 9 (7%) | 0.92 (0.79-0.99) | 0.19 |
| Mild cognitive impairment * | 40 | 9 (22%) | 0.98 (0.95-0.99) |  |
| Moderate-to-severe cognitive impairment** | 43 | 14 (33%) | 0.89 (0.79-0.99) |  |
| *According to neuropsychological examinations, Mini-Mental State Examination (MMSE), Montreal Cognitive Assessments (MoCa) >20 or Clinical Dementia Rating (CDR) <2 performed before or at least three months after an episode of delirium.  **According to neuropsychological evaluation, MMSE/ MoCa ≤20, or CDR 2 to 3, performed before or at least three months after an episode of delirium. | | | | |

| **Appendix Table 3**: Likelihood ratios and post-test probabilities for the three scores at all cut-off points. | | | | |
| --- | --- | --- | --- | --- |
| Cut-off points | LR+ | Post-test probability (positive test) | LR- | Post-test probability (negative test) |
| Score 1 (AL-O-A score) | | | | |
| 2 | 3.3 (2.6–4.2) | 0.36 (0.31–0.42) | 0.04 (0.006–0.30) | 0.01 (0.001–0.05) |
| 2.5 | 5.6 (4.0–7.8) | 0.49 (0.41–0.57) | 0.07 (0.02–0.29) | 0.01 (0.003–0.05) |
| 3 | 14.7 (8.2–26.5) | 0.71 (0.59–0.82) | 0.13 (0.05–0.33) | 0.02 (0.01–0.05) |
| 4.5 | 28.9 (11.9–70) | 0.83 (0.67–0.92) | 0.22 (0.12–0.43) | 0.03 (0.02–0.07) |
| 5 | 30.4 (11.2–82.6) | 0.84 (0.66–0.93) | 0.35 (0.22–0.57) | 0.06 (0.04–0.09) |
| 5.5 | 32.8 (10.2–105) | 0.85 (0.64–0.95) | 0.50 (0.33–0.69) | 0.08 (0.05–0.11) |
| 7.5 | 31.8 (7.4–137) | 0.85 (0.56–0.96) | 0.66 (0.52–0.85) | 0.10 (0.08–0.13) |
| 8 | 23.1 (5.1–104) | 0.80 (0.47–0.95) | 0.76 (0.62–0.93) | 0.12 (0.10–0.14) |
| Score 2 (with objective predictors) | | | | |
| 2 | 2.9 (2.3–3.7) | 0.33 (0.28–0.39) | 0.13 (0.04–0.40) | 0.02 (0.01–0.06) |
| 3 | 5.8 (3.8–8.7) | 0.50 (0.40–0.60) | 0.25 (0.13–0.49) | 0.04 (0.02–0.08) |
| 4 | 11.6 (6.23–21.5) | 0.67 (0.52–0.79) | 0.33 (0.20–0.56) | 0.05 (0.03–0.09) |
| 5 | 16.5 (7.6–35.8) | 0.74 (0.57–0.86) | 0.39 (0.25–0.61) | 0.06 (0.04–0.09) |
| Score 3 (with predictors grouped by CAM features) | | | | |
| 1.5 | 2.1 (1.75–2.4) | 0.27 (0.23–0.29) | 0.06 (0.01–0.41) | 0.01 (0.01–0.07) |
| 2 | 12.1 (6.3–23.3) | 0.68 (0.52–0.80) | 0.36 (0.22–0.59) | 0.06 (0.04–0.09) |
| 3.5 | 12.2 (6.1–24.5) | 0.68 (0.51–0.81) | 0.43 (0.28–0.65) | 0.07 (0.05–0.10) |
| 4 | 17.3 (5.90–50.4) | 0.75 (0.50–0.90) | 0.63 (0.50–0.84) | 0.11 (0.08–0.13) |
| 5.5 | 21.2 (6.3–71.8) | 0.79 (0.52–0.92) | 0.67 (0.52–0.86) | 0.12 (0.08–0.13) |

| **Appendix Table 4**: Inter-rater agreement for study questionnaire predictors. Based on 30 patients examined twice by two independent raters. | | |
| --- | --- | --- |
| Variables | Agreement | Kappa |
| Subjective observation |  |  |
| Normal alertness versus other | 87 | 0.52 |
| Fluctuation of mental state | 87 | 0.53 |
| Illogical flow of ideas | 93 | 0.81 |
| Off-target responses | 93 | 0.76 |
| Tasks/objective observation |  |  |
| Backwards digit test | 74 | 0.45* |
| 3 or more mistakes in backwards digit test | 73 | 0.47* |
| Temporospatial orientation test | 91 | 0.77* |
| Score |  |  |
| **Score 1 (AL-O-A)** | - | - |
| Low risk versus other categories | 87 | 0.72 |
| Any category | 88 | 0.70* |
| **Score 2 (objective predictors)** | - | - |
| Low risk versus other categories | 63 | 0.32 |
| Any category | 75 | 0.46* |
| * weighted kappa | | |

| **Appendix Table 5**: Factors precipitating patient delirium (n = 32). | |
| --- | --- |
| **Factor** | **N (%)*** |
| Infection | 23 (72%) |
| Trauma-pain | 3 (9%) |
| Drugs – (opioids, benzodiazepines, other) | 11 (35%) |
| Urinary retention | 0 |
| Fecal impaction | 1 (3%) |
| ICU | 7 (22%) |
| Primary neurological disease | 2 (6%) |
| Stroke | 3 (9%) |
| Head trauma - neurosurgery | 2 (6%) |
| Toxic, metabolic | 5 (16%) |
| Alcohol, drugs withdrawal | 4 (13%) |
| Epilepsy | 3 (9%) |
| Surgery | 5 (16%) |
| * Multiple precipitating factors are often found in patients with delirium. | |

**
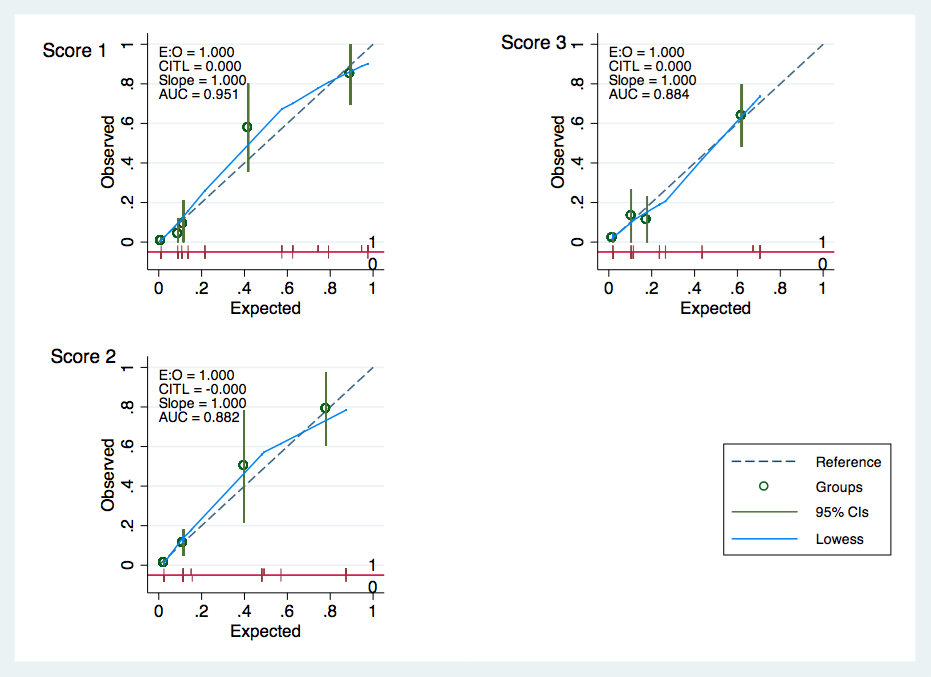
**

**Appendix Figure 2**: Calibration curves for the three scores with LOWESS smoother.

The AL-O-A score

***Al****ertness, temporospatial* ***O****rientation, and off-target* ***A****nswers*

Delirium screening in acute internal medicine

| *For each category, choose the most appropriate answer from I to IV (only one per category). The score is calculated by adding up the points from the three categories or, as a rule of thumb, by counting the categories with mistakes.* | | | | | | |
| --- | --- | --- | --- | --- | --- | --- |
|  | | Answers | | | Points | Category |
| **Alertness:** During the medical interview, the patient is | | I | Calm and awake | | 0 | **A** |
|  |  | II | Drowsy* | | 2.5 |  |
|  |  | III | Agitated ^†^ | |  |  |
|  |  | IV | Fluctuating ^‡^ | |  |  |
|  | | | | | + | **+** |
| **Orientation (**ask):  - **“What is the date today?”** (count one mistake per wrong answer for the day, the month and the year)  - **“Where are we?”** (count one mistake per wrong answer for the state/city and type of building (hospital)) ^§^ | | I | Correct | | 0 | **B** |
|  |  | II | 1 mistake | | 2 |  |
|  |  | III | 2 mistakes | | 2.5 |  |
|  |  | IV | 3 or more mistakes | | 3 |  |
|  | | | | | + | **+** |
| **Answers:** During the medical interview, when asking open-ended questions (needing more than a binary “yes or no” answer) the patient gave | | I | Coherent and appropriate answers | | 0 | **C** |
|  |  | II | Off-target responses^\|\|^ | | 2.5 |  |
|  | | | | | = | **=** |
|  | | | | **Total** ** |  |  |
|  | | | | | | |
| **Low risk**  < 1% risk of delirium | **Intermediate risk**  10% risk of delirium | | | **High risk**  > 80% risk of delirium | | |
| All correct answers  (0 points) | Incorrect answer in one category only (1–3 points) | | | Incorrect answers in 2 or more categories (> 3 points) | | |

# Note that this scale was developed and evaluated in a French-speaking Swiss sample population. The English version provided here has not yet been formally tested.

# * The patient falls asleep during the medical interview and needs stimulation to be roused (voice, loud voice, touch or shaking). First awakening (when entering the room) is not abnormal unless the patient still falls asleep during the medical interview. † The patient has psychomotor agitation, seems inappropriately aggressive, shows excessively strong responses to ordinary objects/stimuli in the environment, or is restless. ‡ The patient displays significant variations in attention or alertness during the interview (or during the day), or has a fluctuating mental state (alertness or level of attention). § Add the number of incorrect answers from the two questions. || The answers are rambling, off-target (only minimally related to the question). ** The score is a screening tool and lacks several essential criteria for a definite diagnosis of delirium, as listed in the Diagnostic and Statistical Manual of Mental Disorders-V. For a diagnosis of delirium, the disturbance must develop over a short period (usually hours/days) and be a direct physiological consequence of a medical condition. Furthermore, disturbances should be recognised as not being better explained by another neurocognitive disorder and should not occur in the context of a severely reduced level of arousal, such as coma.

V 2 : 01.11.2020
